# Simple Aesthetic Sense and Addiction Emerge in Neural Relations of Cost-Benefit Decision in Foraging

**DOI:** 10.1038/s41598-020-66465-0

**Published:** 2020-06-15

**Authors:** Ekaterina D. Gribkova, Marianne Catanho, Rhanor Gillette

**Affiliations:** 1 Neuroscience Program, University of Illinois at Urbana-Champaign, Champaign, United States; 2 Department of Bioengineering, University of California, San Diego, United States; 3 Department of Molecular and Integrative Physiology, University of Illinois at Urbana-Champaign, Champaign, United States

**Keywords:** Computational neuroscience, Motivation, Neural circuits, Reward, Sensory processing, Computational models, Computational biology and bioinformatics, Evolution, Neuroscience, Psychology, Zoology

## Abstract

A rudimentary aesthetic sense is found in the stimulus valuations and cost-benefit decisions made by primitive generalist foragers. These are based on factors governing personal economic decisions: incentive, appetite, and learning. We find that the addictive process is an extreme expression of aesthetic dynamics. An interactive, agent-based model, ASIMOV, reproduces a simple aesthetic sense from known neural relations of cost-benefit decision in foraging. In the presence of very high reward, an addiction-like process emerges. A drug-like prey provides extreme reward with no nutritive value, initiating high selectivity and prolonged cravings for drug through reward learning. Varying reward experience, caused by homeostatic changes in the neural circuitry of reward, further establishes the course of addiction, consisting of desensitization, withdrawal, resensitization, and associated changes in nutritional choice and pain sensitivity. These observations are consistent with the early evolution of addiction mechanisms in simple generalist foragers as an aesthetic sense for evaluating prey. ASIMOV is accessible to inspection, modification, and experiment, is adaptable as an educational tool, and provides insight on the possible coevolutionary origins of aesthetics and the addiction process.

## Introduction

The aesthetic sense is a subjective, evaluative faculty used to distinguish positive and negative qualities of situations, objects, and constructs, and to bias behavioral decision toward or away from those stimuli. It is based on built-in preferences and feature detection, as well as learned preferences established from experience through reward learning. In humans, the highly developed aesthetic sense extends from judgements of taste and beauty to disgust. In other animals, it notably functions in mate choice, nest building, and foraging.

Darwin and others^1,2^ attributed the origin of the aesthetic sense to mate choice and reproductive displays, as are notable in many vertebrates. However, here we explore the ramifications of a potentially earlier origin in the foraging decisions of generalist animal species, where valuations of potential prey are made in estimates of nutritional value that factor in need, learned attributes, and risk. A primitive basis of the aesthetic sense appeared in our studies of the neuronal circuitry of decision in the predatory, generalist sea slug, *Pleurobranchaea californica*, in the animal’s ability to evaluate stimuli in contexts of motivation and reward learning. It was initially implemented in an agent-based foraging simulation, Cyberslug^3^. That agent made foraging decisions for approach or avoidance like the real animal, based on stimulus quality, motivation, and reward learning, and satisfied requirements of optimized foraging.

We introduced the original simulation as an example of simple neuronal relations that could be elaborated for more complex cognition and behavior, as may have happened to ancestral bilaterians in evolution. Accordingly, here we introduce a new version, ASIMOV, which is upgraded for more realistic expression of aesthetic sense with 1) explicit representations of dynamic hedonic tone in reward experience and of noxious pain with direct accesses to the agent’s appetitive state, and 2) a novel mechanism of homeostatic plasticity that contributes to use-dependent desensitization of the reward experience. ASIMOV implements two key forms of plasticity characteristic of the natural aesthetic sense. First, reward learning, which can establish complex preferences to guide acquisitive, synthetic, and creative behaviors. Second is use-dependent habituation to the reward experience produced by repeated exposure to a stimulus. For instance, for animals given access to an unlimited supply of a new and highly palatable food, the relative palatability of that food may decline^4^. Congruently, attention to acoustic and visual stimuli is balanced between repetition/regularity and novelty^5^.

We found in the simulation that the dynamic process of addiction to high reward stimuli emerges as an extreme expression of aesthetic dynamics. Addiction begins with simple reward learning, but the course of addiction through desensitization, withdrawal, and resensitization, with associated changes in nutrition and pain sensitivity, is established through homeostatic changes in the neural circuitry that expresses reward experience. ASIMOV, with its simple homeostatic reward circuit, also reproduces the dynamics of earlier important models of addiction, such as the opponent-process model^6^ where the direct reward input stimulation in ASIMOV can be considered as a primary hedonic process, and the homeostatic plasticity in its reward circuit is analogous to the opponent hedonic process. The results support the view that addiction involves unusually large, rewarding stimuli, to which the forager is not adapted and becomes impaired in its volition.

The ASIMOV model is broadly accessible to inspection, modification, and experiment, is easily adaptable as an educational tool, provides insights on the possible evolutionary origin of the addiction process, and may be developed further for more complex aesthetic tasks.

## Methods

ASIMOV (Algorithm of Selectivity by Incentive, Motivation and Optimized Valuation) derives from a previous simulation^3^ based on reward learning and motivation, and founded on neuronal relations used in cost-benefit choices of foraging by the predatory sea-slug *Pleurobranchaea*. A simple aesthetic sense is expanded in ASIMOV with a homeostatic reward circuit expressing reward experience. Simulating the aesthetic sense requires mechanisms for preference formation by reward learning, and for initiating and terminating preference-seeking behaviors by modulating appetitive state via incentive, reward experience, and satiation. With these, the addiction process emerges in an extreme expression of aesthetic preference.

The forager encounters two virtual prey in the environment, the benign Hermi and noxious Flab (based on natural prey of *Pleurobranchaea*), and a high-reward Drug that provides no nutrition. Each prey and Drug secrete their own signature odors. The ASIMOV forager’s simple aesthetic sense is altered with reward learning, as by experience it associates different signature odors with positive and negative expected rewards, establishing a set of dynamic preferences. The addiction process occurs as exaggerated preference for a high-reward item with a specific odor. For the forager, odor signature is the context in which drug is acquired, analogous to place preference in humans for the site where a drug is obtained, how it is ingested, or the company of like-minded acquaintances. ASIMOV is a minimalist model without critical conditional statements, in which decision emerges at thresholds attained by interactions of variables. This approach is aimed to conservatively model the primitive functionality of aesthetic sense in a simple forager.

### ASIMOV model architecture

**Figure 1:**
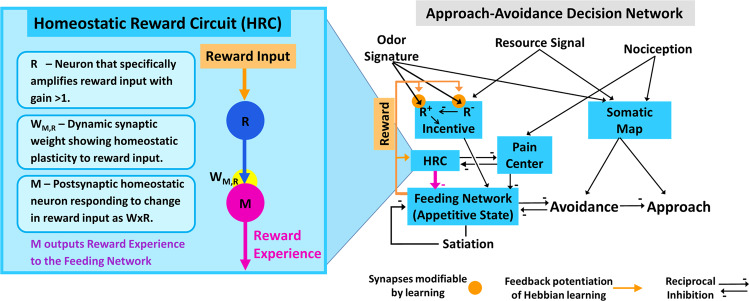
ASIMOV’s neural network of foraging decision. *Right:* In the modified decision network from Cyberslug^3^, Appetitive State (feeding network excitation) sums intrinsic and learned stimulus values as Incentive with motivation (Satiation) to regulate turn response direction. In parallel, a map of stimuli (Somatic Map) from the animal’s oral veil sets turn amplitude. Incentive sums sensory inputs predicting nutritional value (Resource Signal) with learned positive and negative values of prey odor signatures (R + and R-), and is then integrated with motivation, Reward Experience, and Pain into Appetitive State (Feeding Network excitation). Positive and negative classical learning occur by outputs from the feeding network operating in approach or avoidance modes, respectively. *Left:* The Homeostatic Reward Circuit (HRC) mediates habituation to rewarding cues, the basis of Drug desensitization and withdrawal. HRC integrates rewards from prey consumption as Reward Experience and reduces Appetitive State in negative feedback. HRC comprises two connected rate-based neurons, R and M. R receives and amplifies reward output from Feeding Network. Neuron M expresses homeostatic plasticity, habituating to reward. M’s activity is a product of the dynamic synaptic weight W and neuron R’s activity. HRC activity lies in reciprocal inhibition with the Pain Center output; higher levels of pain suppress Appetitive State and cause aversion to painful stimuli. Thus, pain’s suppressive effect is reduced by positive reward output from HRC.

ASIMOV’s structure is shown in Fig. 1. The core of the model is the origin of behavioral choice in appetitive state, which controls economic decisions of foraging^3^. Appetitive state represents the animal’s biases towards appetitive behaviors, including prey tracking, handling, and consumption. By integrating reward experience, incentive, pain, and motivation, appetitive state controls the choice of an approach or avoidance turn, such that low appetitive state causes aversive responses to stimuli, and increasing appetitive state inverts turn response direction to one of approach^7^. Thus, appetitive state sets sensory thresholds for approach turns toward prey and subsequent feeding responses; in switching, excitatory sensory input is routed from one side of the turn network to the other, to cause a turn towards the stimulus.

Sensory inputs are: 1) a resource odor signal predicting nutritional content; 2) specific odor signatures for different prey species; 3) a place code for the averaged site of sensory input to the sensors (Somatic Map); and 4) nociception. (1) and (2) are summed as Incentive for resource and learned positive and negative values of prey odors (R^+^ and R^-^, respectively). Incentive is then integrated with motivation, reward experience, and pain as Appetitive State in the Feeding Network. The sensory place code in Somatic Map acts as a template for turn response amplitude. Positive or negative learning are consequences of feedback from the feeding network operating in feeding or avoidance modes, respectively. Outputs of Appetitive State are reward and a converting function that switches the turn response to stimuli from avoidance to approach.

Reward experience is the output of a homeostatic reward circuit (HRC) module (Fig. 1, left), resulting from reward circuitry activation, that feeds back to Appetitive State. HRC actions resemble habituation^8^. Reward input, as from a recreational drug, is amplified by neuron R and fed to postsynaptic neuron M, whose activity is the product of synaptic weight W and neuron R activity. The synaptic weight W between neurons R and M changes dynamically based on both presynaptic and postsynaptic activity, as well as baseline activity. With repeated or long enduring large reward stimuli, as in Drug reward, homeostatic plasticity desensitizes neuron M’s response, which reduces positive reward effects (such as Drug reward) and causes them to decay faster. Thus, reward experience, a function of neuron M activity, differs with consumption of different prey, and changes drastically with rewarding Drug or withdrawal. If intake of rewarding prey or Drug is relatively frequent, reward experience diminishes due to homeostatic plasticity. Negative reward experience results from noxious prey consumption, and more severely, from Drug withdrawal.

Pain suppresses appetitive state, biasing decision towards avoidance. Pain and reward experience are opponent processes^9^ that are reciprocally inhibitory (Fig. 1, left). Thus, reward experience also influences appetitive state by gating pain input. Each by itself at high values suppresses appetitive state; when either mode dominates, it becomes the major suppressor of appetitive state. Positive reward experience attenuates aversive responses by opposing suppression of appetitive state by pain. However, if reward experience is quite high, then a pain stimulus can evoke an approach turn in the forager by relieving suppression of Appetitive State by reward experience. The model predicts that with positive reward experience, as from Drug consumption, an extremely hungry animal may attack severely painful stimuli.

### ASIMOV simulation

Quantitative results from ASIMOV are obtained by controls on the interface console (Supplementary Methods). These can set prey and Drug populations, variables of satiation and reward experience, and apply pain in controlled settings.

Prey and drug selectivities were examined under “Drug-Free” and “Addicted” states. Specifically, for the Drug-Free state, associative strengths for Flab and Hermi were adjusted to maximums of 1 by pre-feeding the forager 15 of each prey. For each trial, Fixation of Variables was used to set Satiation and Reward Experience to specific values, and then, using Presentation Mode, Flab, Herm, and Drug were separately presented to the forager to test whether the forager made an appetitive or aversive turn. Satiation was set at values ranging from 0.01 to 1.0 and Reward Experience was set at values ranging from −20 to 20. For the Addicted state, the procedures were similar, except that associative strength for Drug was also set at a maximum of 1.

In Addiction Cycle Mode, the user observes the forager in different phases of the addiction processes, where availability of Drug changes over time, starting with only prey and no Drug, and then adding and removing the Drug. In the last phase, Drug is present with its odor signature, but does not provide any reward to test the effects of learning extinction.

ASIMOV is implemented in the graphic, agent-based programming language, NetLogo^10^, and is available at https://github.com/Entience/ASIMOV.

## Results

### Effects of satiation and reward experience on prey and drug selectivity

**Figure 2:**
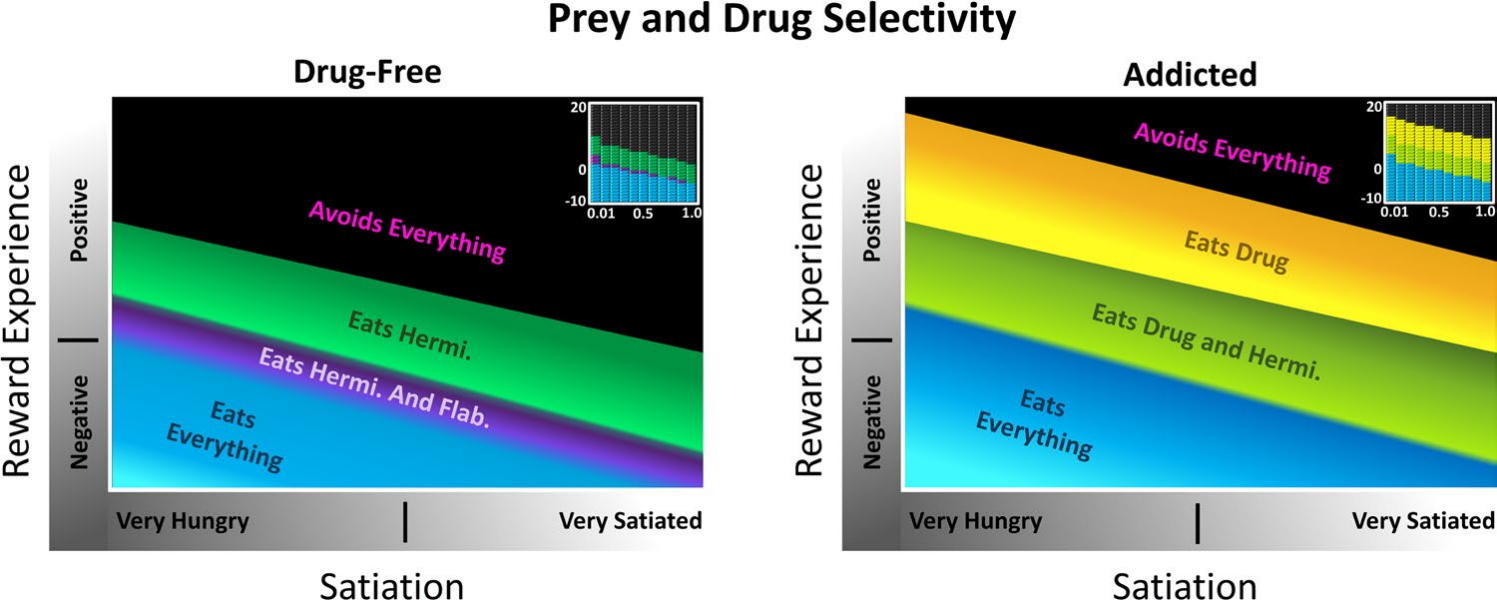
Effects of satiation and reward experience on selectivity for prey and Drug. Relations are smoothed from coarser quantitative data (insets), where satiation is varied from 0.01 to 1.0, and reward experience is varied from −10 to 20 in Presentation Mode. With enough available Drug in the environment, Drug consumption is favored over prey, leading to a lower nutritional state and low satiation. Selectivity is observed as an approach turn towards specific prey or the Drug. *Left*: A selectivity map for ASIMOV’s forager naïve to the Drug. Learned associations for benign Hermi and noxious Flab are at maximum associative strength in this environment, with no learned association for the Drug. As prey consumption provides relatively small positive and negative reward experience, reward experience level is largely near zero. *Right*: An approach turn selectivity map for a forager addicted to Drug. Learned associations for all prey and Drug are at maximum associative strength. Drug consumption gives immediate positive reward and can eventually lead to negative reward experience during withdrawal; thus the forager’s reward experience ranges from negative to positive. In negative reward experiences, like withdrawal, the effect of hunger is increased, and the forager shows less selectivity for prey and Drug. In high positive reward experience, there is increased selectivity for the Drug, so nutritional needs are often ignored in favor of Drug consumption.

ASIMOV’s simple aesthetic sense is modulated by reward experience and satiation. Figure 2 shows the effects of satiation and reward experience on the forager’s selectivity for prey and Drug, under both Drug-Free state and Addicted states (Methods). Notably, under both states, high positive reward experience and high satiation both suppress preference-seeking behavior, as the forager avoids all prey and Drug at the highest levels. This satisfaction of preferences ends preference-seeking behavior. In the context of addiction, withdrawal manifests as negative reward experience, which only affects appetitive state and is a direct consequence of homeostatic plasticity in reward circuitry. The immediate effect of Drug consumption is positive reward experience. Consumption of either prey, noxious Flab or benign Hermi, increases satiation and provides relatively small negative or positive rewards, causing the forager to maintain a “normal” range of reward experience; whereas the Drug causes significant fluctuation in reward experience, as upon consumption it causes immediate extreme increase in positive reward experience, and over time can lead to negative reward experience due to desensitization.

In the Drug-free state, where the forager is not exposed to Drug but learns associations of benign and noxious prey, low satiation leads to decreased selectivity for prey, while higher satiation leads to greater selectivity for benign prey, Hermi. As only prey are available for consumption in a Drug-free state, the forager maintains a normal range of reward experience.

If the forager learns all associations for prey and Drug (Fig. 2, Addicted), selectivity between prey is similar to the Drug-free state. However, since Drug is valued more than all prey, Drug selectivity is enhanced even at high levels of satiation. During negative reward experience, like withdrawal, non-selective consumption of prey is increased, leading to increased satiation and in turn greater selectivity towards Drug. With high positive reward experience, the effect of satiation on appetitive state increases, thereby enhancing selectivity for Drug. Thus, nutritional needs are ignored in favor of Drug consumption. When satiation and reward experience are high enough, the forager becomes averse to all prey and Drugs until either state drops to a lower, permissive value.

**Figure 3:**
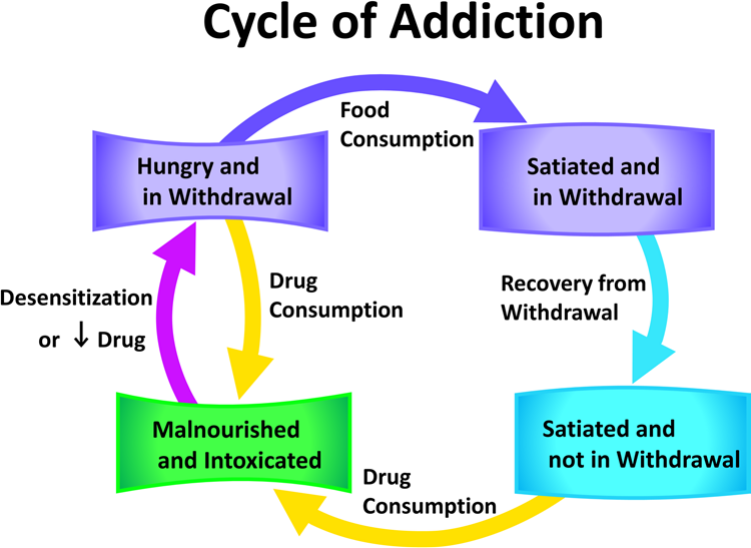
Four general states of ASIMOV’s forager in addiction. With enough food and learned associations for prey and Drug, the cycle leads to Drug seeking, consumption, and poor nutritional state. In withdrawal, hunger has a stronger effect on appetitive state, reducing selectivity for Drug and prey consumption. In this dual state of hunger and withdrawal, the forager can consume the Drug non-selectively, which, if easily available, leads to intoxication and malnourishment. Without Drug it can consume prey non-selectively, increasing satiation. As the forager becomes satiated and recovers from withdrawal, satiation increases its selectivity for the Drug. If enough Drug is available and consumed, the forager’s high positive reward experience reduces the effects of hunger to leave it in a state of malnourishment and intoxication. In this high reward experience, selectivity for the Drug is still increased (see Fig. 2, right), and if sufficient Drug is available, consumption continues to maintain high reward experience. If insufficient Drug is available, or if the forager is too desensitized to Drug reward, it falls into withdrawal, begins feeling the effects of hunger more acutely, and starts the behavioral cycle over again.

Figure 2 thus examines the forager’s dynamic aesthetic sense, showing shifts in preferences across differing satiation and reward experience, as well how these preferences change after a new experience. The more specific and severe instance of the aesthetic process in addiction was further explored. Figure 3 depicts the ASIMOV forager’s generalized states in the Addicted state, where if enough food is available, it enters a cycle of Drug-seeking behavior. Since high satiation increases selectivity for Drug, and withdrawal does not deter Drug consumption, without intervening circumstances the forager inevitably seeks out the Drug when its signature odor is present. So, available Drug naturally leads to high Drug consumption rates and lower nutritional state. Conversely, inadequate Drug supply leads to withdrawal and a period of overconsumption of prey.

More significantly, however, the graphs of Fig. 2 indicate that the forager’s strong preference for Drug is largely independent of fluctuations in reward experience and satiation. In the Addicted state the forager approaches Drug at most levels of satiation and reward experience, and its selectivity for Drug is higher overall than for any prey type. Thus, the primary driver for Drug consumption is the high associative strength and the resulting strong selectivity for Drug.

### Phases of addiction in a dynamic environment

**Figure 4:**
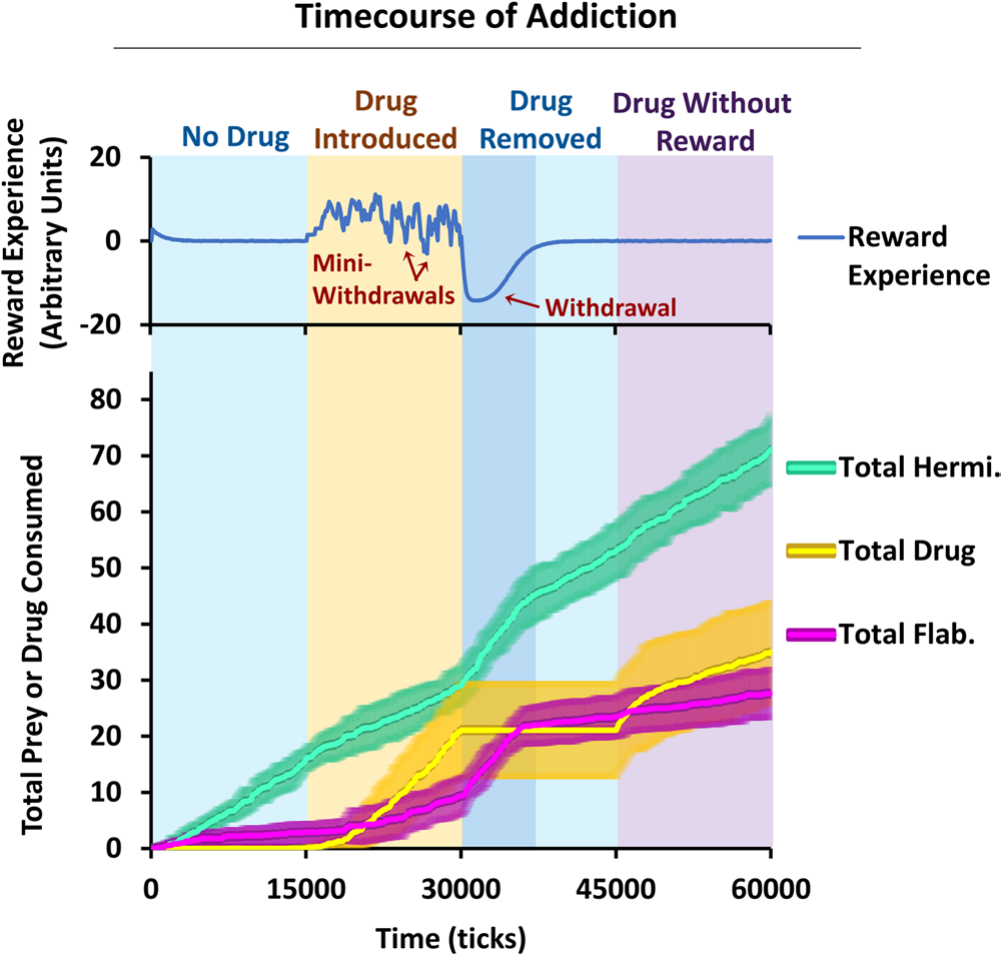
Phases of addiction. In Addiction Cycle Mode, Drug is introduced and removed. In these phases, the forager experiences desensitization, withdrawal and cravings. The curves displayed are timecourses of reward experience and the total numbers of different prey or Drug consumed, averaged over 10 trials of the Addiction Cycle Mode simulation. Error bars (SEM) are shown for total Hermi, Drug, and Flab consumed. When Drug is introduced, first-time Drug consumption typically occurs by accident or at low satiation. As Drug consumption continues, the forager experiences mini-withdrawals, wherein reward experience swings rapidly between positive and negative values. When Drug is removed, the forager undergoes withdrawal, during which loss of reward exacerbates the effects of hunger, increasing non-selective consumption of prey. In the Drug Without Reward phase, when Drug is first reintroduced with the same odor signature but no reward, the forager quickly resumes Drug consumption, even after recovery from withdrawal, since the associative strength for the Drug (“cravings”) is still high. In later stages of Drug Without Reward phase, Drug consumption is significantly reduced due to the decrease in Drug’s associative strength.

The dynamics of the ASIMOV forager’s aesthetic sense and its effect on foraging were explored further in a changing environment, without fixation of variables, using the Addiction Cycle Mode (Fig. 4; Methods, Supplementary Methods).

Here, the environment starts with 3 Flab and 3 Hermi, which respawn in random sites when eaten. Reward experience begins as positive as the forager learns associations for Hermi and Flab, then declines by homeostatic mechanisms. In the second phase, 6 Drug items are introduced. The forager finds and consumes the Drug, typically by accident when attempting to consume nearby prey. After first encounters, Drug consumption rate rises quickly. This coincides with a slight initial decrease in Hermi consumption, likely due to the decreased effects of hunger on the forager’s appetitive state and competition with Drug. As frequent drug consumption continues, desensitization to Drug reward increases, with marked fluctuations and decreasing average reward experience. Thus, in an extended period of drug consumption, especially if Drug is limited, the forager begins to experience “mini-withdrawals”, where rapid switches occur between positive reward experience and brief periods of negative reward experience (Fig. 4). Mini-withdrawals are caused by the forager’s inability to consume Drug quickly enough to overcome the marked desensitization developed in its HRC circuit, causing a brief negative reward experience. In this phase, the forager can markedly change its eating habits, oscillating from overeating prey in withdrawal to low nutrition during high reward experiences.

With removal of Drug, the forager enters withdrawal, followed by slow recovery. In withdrawal, consuming both Hermi and Flab increases markedly due to reduced prey selectivity. Notably, if Drug is reintroduced, consumption is resumed often more quickly than when Drug was first introduced, reflecting the effect of cravings for the drug. As Drug is reintroduced with no reward (Fig. 4, Drug Without Reward), consumption quickly resumes at an initially high rate, which declines as the associative strength between Drug odor and reward decreases with extinction, approaching zero. This post-addiction phase is then similar to the initial Drug-free phase (Fig. 2), and the Drug consumption rate becomes like that for a “neutral” prey without either nutrition or reward, and for which the forager has no associative strength. This suggests that the Drug consumption at the end of the phase is primarily due to accidental consumption and the effects of hunger, rather than a learned association.

### Effects of satiation and reward experience on pain threshold

Pain modifies the effect of reward experience on appetitive state and thus modulates the aesthetic sense. Since pain and reward experience are reciprocally inhibitory, a strong pain stimulus overrides the aversive effect of high reward experience to become the primary aversive influence. This effectively alters the impact of reward experience. A positive reward experience can reduce the effect of pain and thus increases appetitive state, instead of reducing it as happens without pain. In contrast, negative reward experience aggravates the effect of pain.

**Figure 5:**
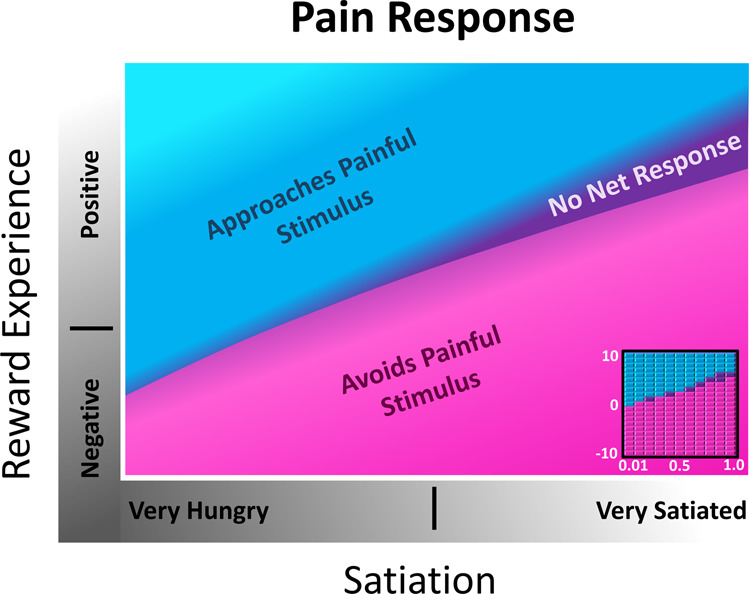
Effects of satiation and reward experience on response to pain. In general, positive reward reduces the ASIMOV forager’s aversion to pain, while negative reward enhances it. Net responses to pain application are classified as approach turns to the stimulus, no net turn, or avoidance turns. Relations are smoothed from coarser quantitative data shown in the inset at the bottom right corner, where satiation is varied from 0.01 to 1.0, and reward experience is varied from −10 to 10 in the simulations. In a neutral reward experience, the forager makes an approach turn towards pain at very low satiation, and avoidance turns at higher satiation levels. A positive reward experience reduces the effect of pain, causing approach turns towards pain at higher satiation levels, while negative reward experience causes aversive turns at all levels of satiation.

To explore the approach-avoidance response to painful stimuli at different values of reward experience and satiation, in Presentation Mode ASIMOV’s forager is immobilized but retains free turning responses, and pain stimuli are applied to the forager’s right anterior region at a strength of 10. Reward experience affects pain thresholds for the aversive turns (Fig. 5). Immediate rewards from Drug consumption effectively increase pain threshold, while withdrawal from Drug lowers it. At very low satiation and without reward, pain induces approach turns, but at a higher state of satiation pain causes avoidance. However, a positive reward experience immediately following Drug consumption reduces the effect of pain and causes approach turns even at even higher satiation levels. Negative reward experience, as occurs in withdrawal, worsens the effect of pain and causes aversive turns at all levels of satiation.

## Discussion

ASIMOV reproduces a simple aesthetic sense, based on known neural circuitry of cost-benefit decision in foraging. Aesthetic valuation is a basic function in the ancient circuitry of foraging behavior, where generalist foragers establish preferences and aversions to the sensory signatures of different prey through reward experience. The aesthetic sense produces affective valuations that are expressed in behavior by characters of approach or avoidance. The signature stimuli connected with different prey acquire salience from interactions of reward learning and motivation, and confer ability to discriminate prey based on rewarding characters.

Reward learning allows opportunistic, foraging generalists that hunt in unpredictable environments to exploit prey available at different times and endowed with special qualities of nutrition or defense. Motivation acts with reward learning to organize cost-benefit analysis of predatory attempts, facilitating the negotiations of risk with need in foraging^3,7^. Reward learning likely has ancient origins, and is documented among generalist foragers in annelids, mollusks, insects, spiders, and even nematodes and flatworms. In parallel are aspects of the aesthetic sense in terms of abilities to evaluate stimuli, and specifically addictive behaviors^11–19^.

The present results are consistent with addiction as an extreme expression of aesthetic choice. Strong learned association between Drug context (here odor signature) and high reward establishes preference for Drug at most values of satiation and reward experience (Figs. 2 and 3). The high-reward Drug stimulus causes strong association between reward context and actual reward. Lower reward stimuli, like the prey Hermi, cause milder effects because they produce a weaker learned association and less fluctuation in reward experience. The lesser effects are better seen as lower level “preferences”, rather than addictions. Thus, while fluctuating reward experience acts together with learned context to influence Drug consumption in addiction, the major factors are learned association and homeostatic plasticity. For an addicted animal, the model predicts that high drug availability leads to high drug consumption rates and lesser nutritional state, as is not unusual in actual drug addiction. In contrast, for a drug-naïve animal, drug consumption is less likely to occur under most circumstances but can be mediated either by chance or external agency (such as through peer pressure).

Corollaries of the addictive experience are desensitization to the rewarding properties of the addictive stimulus, withdrawal, a slow resensitization to drug reward, and prolonged cravings. Homeostatic plasticity, a use-dependent compensatory adjustment in the excitability of neurons and their networks^20^, is strongly implicated in the addiction process, as when drugs affect action potential production or synaptic strengths. The “reward experience” is modified by homeostatic plasticity and thereby accounts for the dynamics of aesthetic valuations and characteristics of addiction. Homeostatic plasticity in ASIMOV’s HRC module is responsible for desensitization and withdrawal by its use-dependent negative feedback to appetitive state. A likely HRC analog in the mammalian brain is the nucleus accumbens, which receives rewarding dopaminergic input, can suppress feeding via GABAergic projections to the appetite center in the lateral hypothalamus^21^, and expresses notable homeostatic plasticity in the addiction process^22^. While the negative affect of withdrawal is associated with reduced dopaminergic signaling in response to reward, it may also involve increased sensitivity of stress systems in extended amygdala, habenula, and hypothalamus^23^.

**Figure 6:**
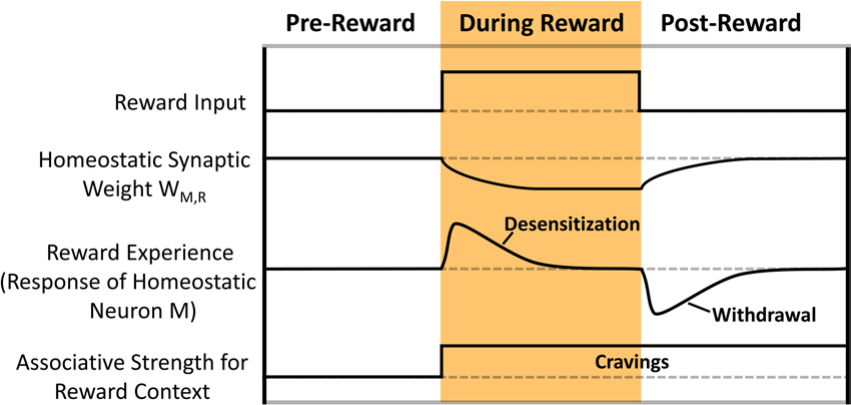
Effects of homeostatic plasticity in addiction. During reward input like Drug consumption, synaptic weight W changes dynamically based on the activity of neurons M and R (q.v. Fig. 1). The response of neuron M to reward input desensitizes during prolonged reward input, and markedly decreases in withdrawal after loss of reward input. If reward input is paired with a stimulus such as Drug odor, after cessation of reward input the associative strength (incentive) for that stimulus remains high. This causes prolonged cravings for the high reward stimulus after completion of withdrawal.

Figure 6 summarizes effects of reward learning and homeostatic plasticity in addiction. Desensitization induces more Drug seeking to keep reward effects high and to oppose the negative reward experience of withdrawal. In withdrawal, without reward the response of the reward circuitry (neuron M) induces negative affect. Then pain and hunger have greater impacts on appetitive state. If cessation of reward input endures long enough, reward circuitry resensitizes and negative affect of withdrawal decreases. However, the association between reward context, such as Drug odor, and positive reward remains high, resulting in “cravings”: marked increases in appetitive state and approach behavior whenever contextual stimuli (CSs) associated with the high reward (Drug), are encountered, analogous to powerful desire.

What is the adaptive significance of a homeostatically plastic reward circuit in an active forager in a natural environment? HRC, and its negative feedback to the feeding network, could normally function to maintain caloric intake in an environment with changing availability of different food sources. Thus, natural function may be to maintain caloric intake in “feast or famine” scenarios. When prey are readily available, rewarding food items could be common and consumed with high frequency. High frequency consumption would be maintained by desensitization to reward in the HRC, as inhibitory feedback to the feeding network would be reduced. In a famine scenario, if a preferred food source became scarce, ceasing its consumption would lead to some level of withdrawal, resulting in less HRC inhibition of the feeding network and a period of decreased selectivity for all food types, which could adaptively promote caloric intake.

In ASIMOV, desensitization to a repeated, moderately rewarding prey type causes only small withdrawal effects easily managed with consumption of other rewarding foods. Withdrawal from the high-reward Drug is more severe and the same high magnitude of reward would only be available from other addictive agents. Likewise, heroin users in withdrawal may resort to cocaine to alleviate withdrawal symptoms^24^ or other opioid receptor agonists, such as methadone^25,26^. These agonists are also addictive, and withdrawal typically needs further treatment. More broadly, some addictive behaviors have high rates of co-occurrence with substance use, such as gambling^27^, reflecting common neurobiological and molecular pathways^28^. Notably, in ASIMOV withdrawal decreases selectivity among prey (Figs. 2 and 4), potentially allowing the forager to seek novel stimuli that might bring some positive reward.

In ASIMOV’s forager, satiation and reward experience modulate motivation in foraging and seeking behaviors. Both positive reward experience and high satiation suppress appetitive state, thereby promoting avoidance and higher selectivity in foraging. In contrast, negative reward experience and low satiation stimulate approach and lower selectivity. Similarly, the hypothalamic circuits governing hunger and satiety modulate the reward system, where hunger can increase the reinforcement, behavioral responsiveness, and seeking of drugs of abuse, while satiety signals generally reduce these effects^29,30^.

Reward experience and pain have reciprocal effects. Negative reward experience in withdrawal exacerbates effects of both pain and hunger, while positive reward experience (like Drug consumption), can reduce effects of pain and hunger on appetitive state (Figs. 2 and 5). The role of pain in the addiction process begins with the ability of rewarding stimuli and their learned cues to suppress its awareness^31^. This may be adaptive for the foraging animal when dealing with prey defenses (Gillette *et al*., 2000) or perhaps needing to ignore an injury to hunt. Hunger may also inhibit non-acute, inflammatory pain, though hunger itself may be suppressed by acute pain^32^. Pain relief by itself can be rewarding^33^. Desensitization to drugs like cocaine and amphetamines acting on dopaminergic reward pathways can bring on painful side effects, perhaps in part because reward pathways that act to suppress effects of pain^34^ are habituated. More seriously, withdrawal from opiates is worsened, as natural reward mechanisms are blunted and pain pathways simultaneously rebound from drug suppression with overshooting strength likely to also originate in homeostatic plastic mechanisms.

ASIMOV’s forager developed a high rate of Drug consumption whenever it was available (Fig. 4). First-time Drug consumption typically occurred when the forager was in a low satiation state, or by accident as when Drug was very close to nearby prey. When Drug was removed, withdrawal occurred, followed by recovery. When a new version of Drug was then introduced without reward, the forager immediately resumed a high Drug consumption rate, as it retained high associative strength for Drug, representing “cravings”. But, as Drug was consumed without reward, the association for Drug extinguished, and consequently consumption rate fell significantly. These results reinforce the notion that associative strength with reward is a strong driver of addictive drug consumption. They also show that when the recurring context in which the Drug is acquired is paired with non-rewarding or aversive stimuli, it can diminish Drug consumption. This works well in simulation, where control of variables is rigid, but is not easily done in human populations.

### Comparison to other models and theories of addiction

Basic characters of previous theoretical treatments emerge in ASIMOV’s function. Notably, in the opponent-process theory of motivation^6^, hedonic scale and the standard patterns of affective dynamics are analogous to the fluctuations in ASIMOV’s reward experience before, during, and after reward input (Fig. 6). Specifically, 1) in opponent-process the peak of the primary hedonic process theory corresponds to ASIMOV’s reward experience in onset of rewarding input, 2) “hedonic adaptation” corresponds to desensitization of reward experience, 3) “affective after-reaction” corresponds to withdrawal, and 4) “decay of after-reaction” corresponds to resensitization. Moreover, in the simple HRC module of ASIMOV, the primary hedonic process of opponent-process theory relates to neuron R’s response to direct reward input. The secondary opponent hedonic process, which is slow and initiated by the first process, is analogous to homeostatic plasticity of neuron M in the HRC (Fig. 1, left). As in neuronal homeostatic plasticity, the secondary opponent process changes with use, such that rewarding effects are diminished and withdrawal effects increase. This is analogous to the cumulative effects of desensitization via the HRC.

Redish *et al*.^35^ attributed the emergence of addiction to vulnerabilities in decision-making arising in animals’ systems for observation, planning, or habit. For ASIMOV, these correspond to sensory odor integration and the somatic mapping function, appetitive state (including HRC), and the reward learning algorithms, respectively. Two of the primary vulnerabilities obvious in ASIMOV are homeostatic dysregulation and overvaluation, which may have been among the first to emerge in the evolution of mechanisms underlying aesthetic and addictive processes.

Previous computational studies examined addiction through reinforcement learning (RL) models^36,37^, and RL actor-critic models^38^. These did not account for homeostatic processes or the internal state of an organism. The Deperrois *et al*.^39^ model of nicotine addiction used homeostatic down-regulation of receptors in addition to an RL framework; however, it did not account for internal state, and specifically focused on nicotine addiction’s effect on mammalian circuitry. Keramati *et al*.^40^ proposed an actor-critic model of homeostatic reinforcement learning (HRL) combining homeostatic and RL theories of addiction with the effects of organismal internal state, suggesting that rewards calculated by the organism are naturally modulated by its internal state. ASIMOV also takes into account RL, homeostatic mechanisms, and internal state with simpler calculations, in particular for internal state and its integration with learned associations and external stimuli.

Few computational models explore the origins of addiction or impulsivity from foraging circuitry. Barack and Platt^41^ introduced a model of foraging decision comparing values of short-term options against long-term reward rates in iterated foreground-background accept-or-reject contexts, proposing that impulsivity, as seen in addiction, results from inaccurate estimation of long-term reward rates. In an area-restricted search (ARS) foraging model^42^, Hills proposed that too much dopamine signaling was associated with a much too focused cognitive ARS, as may be the case in addiction. ASIMOV differs from these models in its architecture, but both the attentional aspect of ARS and the mis-estimation of long-term rewards resemble ASIMOV’s forager over-learning and over-estimating Drug reward, and becoming extremely focused on attaining and consuming Drug, often ignoring other prey and hunger effects.

Berridge and collaborators described the emergence of addiction in the processes that incentivize rewarding stimuli^43^. These authors differentiated “liking” and “wanting” in the addiction process. Liking describes “in-the-moment” hedonic experiences responding to stimuli, analogous to ASIMOV’s immediate stimulus-driven change in reward experience. Wanting, the motivational drive that lends salience to incentive stimuli, is enhanced by learning of cues. In ASIMOV, wanting is embodied in the relations between the Appetitive State and Incentive modules that determine associative strengths to set the salience of incentivized stimuli (Fig. 1).

### Comparison to other models and theories of aesthetics

Aesthetics relies heavily on attention, as where objects considered beautiful or the opposite by observers will often draw their focus. Thus, aesthetics should also include a liking aspect, and the mere observation or experience of a beautiful object will bring pleasure often not associated with biological urgency^44^. As in addiction, the liking aspect of aesthetics must also depend on a reward system like the HRC, dissociated from physiological need and with internal dynamics independent from external reward input. For instance, the aesthetic pleasure received from observing a painting serves no immediate physiological purpose^44^. Boredom, habituation, and decreasing aesthetic pleasure received from a painting viewed multiple times may be explained by the homeostatic plasticity of a reward system like the HRC, where multiple encounters with the same pleasing stimulus, particularly over a short period of time, decrease the immediate change in reward experience and so decrease the aesthetic pleasure received.

Rolls^45^ explored an origin of aesthetics in goal-directed behavior, suggesting that gene-specified rewards and punishers can establish inherent aesthetic values. But the explicit, rational planning system also affects this valuation, allowing decisions made that might be in the subjective interest of the individual but not necessarily the genes. In ASIMOV, the genetically inheritable analogs include the forager’s general attraction towards betaine, aversion to pain, suppression of appetitive state by satiation, and positive reward experience, and all other relations not included in its reward learning system; this in turn may be considered as the rational system. Both ASIMOV’s genetic and rational systems, while fairly simple, are crucial to decision-making in foraging, contributing to the final integration of appetitive state to specify the forager’s action selection (approach-avoidance).

Xenakis and Arnellos^46^ proposed that aesthetic perception involves interactions where uncertainty is high, with no available relevant knowledge, and where emotions are used to evaluate interactive indications and thereby reduce uncertainty. This is most closely related to ASIMOV’s reward experience, which becomes a major factor in setting appetitive state to make an approach or avoidance decision when the agent is in an uncertain, unfamiliar situation, where it cannot rely on learned or innate associations.

Dissanayake^47^ provides an interesting perspective on evolution of art and aesthetic appreciation from proto-aesthetic operations founded in adaptive ancestral mother-infant interactions. A mother’s vocalizations with her infant, coinciding with increased parental care in human evolution, is an example of a “make special” primitive, where ordinary things are deliberately made significant, and is one of the important ingredients of art. ASIMOV might provide some insight for the origin of even simpler aesthetic primitives. A primitive that should precede a make special primitive entails the existence of specific attention mechanisms. Indeed, reward experience and incentive, and how they modulate appetitive state, involve very simple attentional mechanisms, with homeostatic plasticity of the HRC potentially relating to attentional habituation and sensitization.

To our knowledge there are no other computational models exploring aesthetics in a foraging context. There are computational models of aesthetics and creativity, however these are mainly limited to the field of machine learning and information theory. Schmidhuber^48^, for instance, introduced an intrinsically-motivated agent-based model, and proposed that creativity and associated behaviors could be driven by a simple algorithm using reinforcement learning to maximize an agent’s internal joy to discover and create novel patterns. While ASIMOV does not address novelty seeking, the homeostatic plasticity of the reward system can explain why a reward input that is given repeatedly loses its effect with its novelty. It thus becomes less aesthetically pleasing, revealing characters of boredom and providing a basis for seeking novelty. Thus, ASIMOV might be easily developed further, so that stimulus-specific reward experiences would decline, mimicking boredom and promoting seeking of new aesthetic experiences.

### ASIMOV’s limitations

ASIMOV is a relatively simple model that does not take on all the intricate dynamics of aesthetics and addiction in humans and other mammals. ASIMOV’s forager makes the simplest of decisions for approach or avoidance turns. There are no multi-step decision-making processes and no complex motor output. The Rescorla-Wagner algorithm for learning used is one of the simplest; it does not simulate episodic or sequenced memory and is less complex than the reinforcement learning algorithms employed in other models of addiction or aesthetics. ASIMOV’s architecture is largely based on *Pleurobranchaea*’s circuitry for foraging decisions, with linear and sigmoidal relations between elements, rather than on mammalian learning, reward, and decision circuits modeled with spiking neurons. While this reduces biophysical realism for ASIMOV, it is significant that the minimal model captures common origins of addiction and aesthetics in foraging circuitry. Further expansions of ASIMOV for sequence learning and simple episodic memory may greatly enhance the forager’s aesthetic.

## Conclusion

Addiction emerges as an extreme expression of aesthetic preference. The consequences of the addictive experience are desensitization to the rewarding properties of the addictive stimulus, withdrawal, a slow resensitization to drug reward, and prolonged cravings. Homeostatic plasticity, a use-dependent compensatory adjustment in the excitability of neurons and their networks, is strongly implicated in the addiction process. The modification of the reward experience by homeostatic plasticity thus accounts for the dynamics of both aesthetic valuations and characteristics of addiction. The relations prominent in the ASIMOV simulation – strong preferences, desensitization, withdrawal, resensitization, and protracted cravings – overlap with those that attend the highs and lows of social relationships^49^ and compulsive behaviors like gambling, shopping, internet use, and self-harm^27,50,51^. Reward learning, as well as reward experience and its relationship to pain and modulation by homeostatic plasticity, are causally central to these conditions.

These relations may also lie at the root of innate and learned aesthetic preferences in food, music, and art, as well as the drive behind creative activities. The common relations suggest that the diverse aesthetic processes of affective valuation in higher vertebrate experience are evolutionary derivatives of the basic neuronal circuitry of foraging economics, put to different functions but conserving similarities in their overall organization. Evidence to test this hypothesis is presently scant, but might be usefully sought in comparative studies.

ASIMOV is an easily accessible agent-based simulation, where the decisions and movement of the forager are readily observable, and the interface allows for easy user interaction, including control of the forager’s behavior and environment. The software is highly accessible to verification and experiment, and is available on the internet (https://github.com/Entience/ASIMOV) for examination, use, and modification.

## Supplementary information

Supplementary Information.

## References

[1] Darwin, C. The descent of man and selection in relation to sex. Vol. 1 (Murray, 1888).

[2] Prum, R. O. The Evolution of Beauty: How Darwin’s Forgotten Theory of Mate Choice Shapes the Animal World-and Us. (Anchor, 2017).

[3] Brown, J. W. *et al*. Implementing Goal-Directed Foraging Decisions of a Simpler Nervous System in Simulation. eNeuro **5**, ENEURO. 0400-0417.2018 (2018). 10.1523/ENEURO.0400-17.2018 PMC5830682 29503862

[4] Young PT (1946). Studies of food preference, appetite and dietary habit. VI. Habit, palatability and diet as factors regulating the selection of food by the rat. J. Comp. Psychol..

[5] Berlyne, D. E. Aesthetics and psychobiology. Vol. 336 (Appleton-Century-Crofts New York, 1971).

[6] Solomon RL, Corbit JD (1974). An opponent-process theory of motivation: I. Temporal dynamics of affect. Psychol. Rev..

[7] Gillette R, Huang R-C, Hatcher N, Moroz LL (2000). Cost-benefit analysis potential in feeding behavior of a predatory snail by integration of hunger, taste, and pain. Proc. Nat. Acad. Sci. USA.

[8] McSweeney FK, Murphy ES (2009). Sensitization and habituation regulate reinforcer effectiveness. Neurobiol. Learn. Mem..

[9] Elman I, Borsook D (2016). Common brain mechanisms of chronic pain and addiction. Neuron.

[10] Wilensky, U. NetLogo: Center for connected learning and computer-based modeling. *Northwestern University, Evanston, IL* **4952** (1999).

[11] Palladini G (1996). A pharmacological study of cocaine activity in planaria. Comp. Biochem. Physiol., C: Toxicol. Pharmacol..

[12] Carter K, Lukowiak K, Schenk JO, Sorg BA (2006). Repeated cocaine effects on learning, memory and extinction in the pond snail Lymnaea stagnalis. J. Exp. Biol..

[13] Lee H-G, Kim Y-C, Dunning JS, Han K-A (2008). Recurring ethanol exposure induces disinhibited courtship in Drosophila. PLoS One.

[14] Nathaniel TI, Panksepp J, Huber R (2009). Drug-seeking behavior in an invertebrate system: evidence of morphine-induced reward, extinction and reinstatement in crayfish. Behav. Brain Res..

[15] Heberlein U, Tsai LT-Y, Kapfhamer D, Lasek AW (2009). Drosophila, a genetic model system to study cocaine-related behaviors: a review with focus on LIM-only proteins. Neuropharmacol..

[16] Barron AB, Maleszka R, Helliwell PG, Robinson GE (2009). Effects of cocaine on honey bee dance behaviour. J. Exp. Biol..

[17] Entler BV, Cannon JT, Seid MA (2016). Morphine addiction in ants: a new model for self-administration and neurochemical analysis. J. Exp. Biol..

[18] Shipley AT (2017). The sensitivity of the crayfish reward system to mammalian drugs of abuse. Front. Physiol..

[19] Kusayama T, Watanabe S (2000). Reinforcing effects of methamphetamine in planarians. NeuroReport.

[20] Turrigiano GG, Nelson SB (2004). Homeostatic plasticity in the developing nervous system. Nat. Rev. Neurosci..

[21] Lutter M, Nestler EJ (2009). Homeostatic and hedonic signals interact in the regulation of food intake. The Journal of nutrition.

[22] Huang YH, Schlüter OM, Dong Y (2011). Cocaine-induced homeostatic regulation and dysregulation of nucleus accumbens neurons. Behav. Brain Res..

[23] Volkow ND, Michaelides M, Baler R (2019). The Neuroscience of Drug Reward and Addiction. Physiol. Rev..

[24] Leri F, Bruneau J, Stewart J (2003). Understanding polydrug use: Review of heroin and cocaine co‐use. Addiction.

[25] Amato L, Davoli M, Ferri M, Gowing L, Perucci CA (2004). Effectiveness of interventions on opiate withdrawal treatment: an overview of systematic reviews. Drug Alcohol Depend..

[26] Hirata A, Castro-Alamancos MA (2006). Relief of synaptic depression produces long-term enhancement in thalamocortical networks. J. Neurophysiol..

[27] Walther B, Morgenstern M, Hanewinkel R (2012). Co-occurrence of addictive behaviours: personality factors related to substance use, gambling and computer gaming. Eur. Addict. Res..

[28] Nestler EJ (2005). Is there a common molecular pathway for addiction?. Nat. Neurosci..

[29] Zheng D, de Vaca SC, Carr KD (2012). Food restriction increases acquisition, persistence and drug prime-induced expression of a cocaine-conditioned place preference in rats. Pharmacol. Biochem. Behav..

[30] Cassidy RM, Tong Q (2017). Hunger and satiety gauge reward sensitivity. Front. Endocrinol. (Lausanne).

[31] Altier N, Stewart J (1999). The role of dopamine in the nucleus accumbens in analgesia. Life Sci..

[32] Ponomarenko A, Korotkova T (2018). Hunger is a gatekeeper of pain in the brain. Nature.

[33] Navratilova E, Porreca F (2014). Reward and motivation in pain and pain relief. Nat. Neurosci..

[34] Leknes S, Tracey I (2008). A common neurobiology for pain and pleasure. Nat. Rev. Neurosci..

[35] Redish AD, Jensen S, Johnson A (2008). Addiction as vulnerabilities in the decision process. Behav. Brain Sci..

[36] Redish AD (2004). Addiction as a computational process gone awry. Science.

[37] Dezfouli A (2009). A neurocomputational model for cocaine addiction. Neural Comput..

[38] Takahashi Y, Schoenbaum G, Niv Y (2008). Silencing the critics: understanding the effects of cocaine sensitization on dorsolateral and ventral striatum in the context of an actor/critic model. Front. Neurosci..

[39] Deperrois, N., Moiseeva, V. & Gutkin, B. Minimal Circuit Model of Reward Prediction Error Computations and Effects of Nicotinic Modulations. *Frontiers in neural circuits* **12** (2018). 10.3389/fncir.2018.00116 PMC6336136 30687021

[40] Keramati M, Ahmed SH, Gutkin BS (2017). Misdeed of the need: towards computational accounts of transition to addiction. Curr. Opin. Neurobiol..

[41] Barack, D. L. & Platt, M. L. Engaging and exploring: cortical circuits for adaptive foraging decisions in Impulsivity 163-199 (Springer, 2017). 30351563

[42] Hills TT (2006). Animal foraging and the evolution of goal‐directed cognition. Cogn. Sci..

[43] Berridge KC, Robinson TE (2016). Liking, wanting, and the incentive-sensitization theory of addiction. Am. Psychol..

[44] Mechner F (2018). A Behavioral and Biological Analysis of Aesthetics: Implications for Research and Applications. The Psychological Record.

[45] Rolls, E. T. The Origins of Aesthetics: A Neurobiological Basis for Affective Feelings and Aesthetics in The Aesthetic Mind: Philosophy and Psychology (eds E., Schellekens & P., Goldie) Ch. 8, 116–165 (Oxford University Press, 2011).

[46] Xenakis I, Arnellos A (2014). Aesthetic perception and its minimal content: a naturalistic perspective. Front. Psychol..

[47] Dissanayake E (2015). “Aesthetic Primitives”: Fundamental Biological Elements of a Naturalistic Aesthetics. Aisthesis. Pratiche, linguaggi e saperi dell’estetico.

[48] Schmidhuber J (2010). Formal theory of creativity, fun, and intrinsic motivation (1990–2010). IEEE Transactions on Autonomous Mental Development.

[49] Burkett JP, Young LJ (2012). The behavioral, anatomical and pharmacological parallels between social attachment, love and addiction. Psychopharmacology.

[50] Blasco-Fontecilla H (2016). The addictive model of self-harming (non-suicidal and suicidal) behavior. Frontiers in psychiatry.

[51] Rosenberg, K. P. & Feder, L. C. An introduction to behavioral addictions in Behavioral Addictions 1-17 (Elsevier, 2014).

