# Supplementary material for: Simple Aesthetic Sense and Addiction Emerge in Neural Relations of Cost-Benefit Decision in Foraging
Source: Sci Rep. 2020 Jun 15;10:9627. doi: 10.1038/s41598-020-66465-0 (PMC7295997; doi:10.1038/s41598-020-66465-0)
Supplement: Supplementary file 1 — Supplementary Information. [file 41598_2020_66465_MOESM1_ESM.docx]

Supplementary Information for

**Simple Aesthetic Sense and Addiction Emerge in Neural**

**Relations of Cost-Benefit Decision in Foraging**

Ekaterina D. Gribkova, Marianne Catanho, and Rhanor Gillette

Rhanor Gillette

Email: rhanor@illinois.edu

**This file includes:**

Supplementary methods

Fig. S1

References for supplementary information reference citations

**Supplementary Methods**

The ASIMOV model is derived from the original Cyberslug™ foraging simulation [1] with essential modifications. Cyberslug presents the logic of foraging decision based on reward learning and motivation, and is available at <https://github.com/Entience/Cyberslug>. ASIMOV is implemented in the same graphic, agent-based programming language, NetLogo, version 6.0.4 [2], and is available as extended data at [https://github.com/Entience/ASIMOV](https://github.com/Entience/Cyberfiend). NetLogo software is chosen for its simple availability and accessibility to a broad audience.

The ASIMOV forager encounters two virtual prey in its environment, the benign Hermi and noxious Flab, named after prey sea-slugs *Hermissenda crassicornis* and *Flabellina iodinea* prey that *Pleurobranchaea* encounters in the wild [3]. Each prey secretes two odors: the resource signal *betaine*, a predictor of nutritional resource [4], and either of “*odor_hermi*” or “*odor_flab*.” Odors diffuse realistically over time and space. Prey move in simple random walks. Prey numbers are constant; when consumed, replacements appear at random positions. The specific odors of the prey Hermi and Flab become associated with positive and negative expected rewards, respectively, resulting in learning preference for Hermi and avoidance of Flab. These positive and negative associations are established in reward learning via the Rescorla-Wagner algorithm for classical conditioning [5]. These effects are analogous to the actual predator’s learned preferences for specific prey: ready consumption of the beneficial *Hermissenda*, and the rejection and aversive learning for the noxious Flab [3]. Also present is the analog of an addictive Drug, a high-reward item with its own sensory signature, which provides no nutrition. If prey nor Drug no longer provide reward upon consumption, the corresponding learned association decreases in strength via an extinction mechanism of the Rescorla-Wagner algorithm, in which the received reward is set to zero.

**Appetitive State**

The core of the AIMOV forager model, appetitive state, controls the choice of an approach or avoidance turn, and is a final integration of an animal’s motivational state with stimulus incentive and pain. Motivational state itself is taken as a composite of satiation, a function of nutrition, and feedback from reward experience. By default, when appetitive state is low, the response to stimuli is avoidance, as in actual *Pleurobranchaea* [4]. Increasing appetitive state inverts the turn response direction to one of approach. Thus, appetitive state determines sensory thresholds for the approach turn toward prey and subsequent feeding responses. When high enough, corollary outputs from the feeding network switch the excitatory sensory input of the stimulus from one side of the turn network to the other, resulting in a turn towards the stimulus. Appetitive State is expressed as:

, (1.1)

$S=c_{1}\cdot Incentive-c_{2}\cdot Satiation-c_{3}\cdot Pain-c_{4}\cdot PainSwitch\cdot RewardExperience$, (1.2)

 , (1.3)

where c_1_, c_2_, …, c_6_ are constants available in the code. AppStateSwitch acts as a threshold-based switch between avoidance and approach turning behavior, with (AppStateSwitch - 1) causing a transient suppression of AppState during avoidance turns.

Incentive mixes intrinsic positive and negative properties of stimuli to integrate with memories of previous reward. In absence of incentivized sensory input or pain input, appetitive state is simply the basal motivational state, which in feeding behavior is based on integrating satiation, pain, and reward experience feedback. With addition of incentive, appetitive state becomes equivalent to “incentive salience” as defined in mammals [6].

Satiation determines the baseline excitation state of the feeding network. At very low satiation, appetitive state is high, resulting in an approach turn towards any odor, even if novel. Sensory inputs integrate with memory into incentive. Incentive sums with satiation in the feeding network to either increase or decrease appetitive state.

ASIMOV extends the original model of approach-avoidance decision [1] by adding reward experience (Eq. 2.1), explicit pain sensation, and reciprocal inhibition between them via the PainSwitch variable.

**Reward Experience**

Reward experience, an animal’s activation of reward circuitry under the influence of homeostatic mechanisms, is expressed as a function of the output of the HRC module (Fig. 1, left, and Eqs. 2.1-2.3):

, (2.1)

$M=W\cdot R$ , (2.2)

$\frac{dW}{dt}=\frac{M_{0}-M}{\tau\cdot R}$ , (2.3)

where c_7_ and τ are constants available in the code. In the model (Fig. 1, left), a high reward input, as from a recreational drug, is amplified by neuron R. The amplified reward then feeds to a postsynaptic neuron M, whose capacity for homeostatic plasticity is analogous to habituation [7]. Specifically, the synaptic weight W between presynaptic neuron R and postsynaptic neuron M changes dynamically based on both presynaptic and postsynaptic activity, as well as on baseline activity, M_0_, of neuron M (Eq. 2.3). Neuron M activity is computed as the product of its synaptic weight W and the activity of neuron R (Eq. 2.2), and fed to the Feeding Network.

With repetition of a large rewarding stimulus or a long enduring Drug reward, the reward response of neuron M desensitizes by homeostatic plasticity. Notably, desensitization reduces positive reward effects (such as Drug reward), which also decay faster. Cessation of strong reward causes severe withdrawal. The magnitude of repeated rewards is proportionate to the rate of desensitization and magnitude of withdrawal.

ASIMOV captures the notion of “alliesthesia” [8], the dependence between the internal state of an organism and the perceived pleasure or displeasure of stimuli, in terms of the influence of motivational state on the bare-bones reward experience. We implicitly take the reward experience as an evolutionary precursor of pleasure/displeasure. In humans the aesthetic is involved with “pleasure”. We derive “reward experience” as a dynamic measure of hedonic tone. But the sea slug lacks the cognitive embellishments of declarative and episodic memory. Thus, we use “reward experience” instead of “pleasure”, which is fraught with anthropomorphism. The appropriateness of using “reward experience” also derives from its relation to reward learning, and its potentially better communication with a broader readership.

**Pain**

Pain modifies the effect that reward experience has on appetitive state (Eq. 1.2). Since pain and reward experience are reciprocally inhibitory processes (Fig. 1, left), strong pain stimuli override the general aversive effect of high reward experience to become the primary aversive influence. This reciprocal inhibition is performed via the Pain Switch variable defined in Eq. 1.3, which produces a sign change from +1 to -1, when pain is high. This effectively alters the effect of reward experience on appetitive state. A positive reward experience can actually reduce the effect of pain and thus promote appetitive state, instead of inhibiting it as it does normally. In contrast, a negative reward experience aggravates the effect of pain.

**ASIMOV Interface**

Quantitative results from ASIMOV are obtained by the controls on the interface console (Fig. S1). Important controls on the left side are Prey and Drug Population Controls, Fixation of Variables, Pain Application Controls, and Presentation Mode, and on the right are Addiction Cycle Mode. Prey and Drug Population Controls let the user control the amount of Hermis, Flabs, and Drug present in the environment. Fixation of Variables lets the user fix values for satiation, reward experience, and incentive. Satiation can range from 0.01 to 1.0, reward experience to -20 to 20, and incentive to -10 to 10. The default ranges are adjustable by editing the interface in NetLogo. Pain Application Controls allow the application of a painful stimulus to ASIMOV’s forager. The strength of the applied painful stimulus can be adjusted by the Apply_Pain slider, ranging from 0 to 30 arbitrary units. The Poke-Left and Poke-Right buttons apply pain to the anterior left or right side part of the forager, respectively.

*Presentation Mode* controls the forager’s prey and Drug intake, and tests approach and avoidance responses to specific prey or Drug. The forager can be immobilized except for approach-avoidance turns, and can be force-fed or presented with Hermi, Flab, or Drug to adjust the corresponding associative strengths, or to monitor the effects on reward experience. Presentation with a stationary Hermi, Flab, or Drug near its left side tests its turning response in terms of approach or avoidance towards the prey or Drug.

*Addiction Cycle Mode*, when enabled, allows the user to observe ASIMOV’s forager as it freely forages and experiences different phases of the addiction processes of desensitization, withdrawal, and cravings. In this mode the availability of Drug changes over time, starting with an environment with only prey and no Drug, and then adding and removing the Drug, causing the forager to go through desensitization, withdrawal, and cravings. In the last phase, the Drug is present with the Drug odor signature, but does not provide any reward on consumption. In this phase, Drug consumption decreases significantly (see Fig. 6 for results from the Addiction Cycle Mode). The Addiction Cycle Mode lasts 60000 software cycles (ticks), in which each phase lasts 15000 ticks. During an initial “No Drug” phase, the environment contains only the prey Hermi and/or Flab, letting ASIMOV’s forager learn the corresponding associations. In the second “Drug Introduced” phase, the Drug is introduced for the first time with a high reward on consumption. The third phase is “Drug Removed” and the fourth and last phase is “Drug without Reward”, where the Drug is reintroduced with the same odor signature, but provides no reward on consumption, causing ASIMOV’s forager to decrease its associative strength for the Drug via a Rescorla-Wagner algorithm for extinction.

**
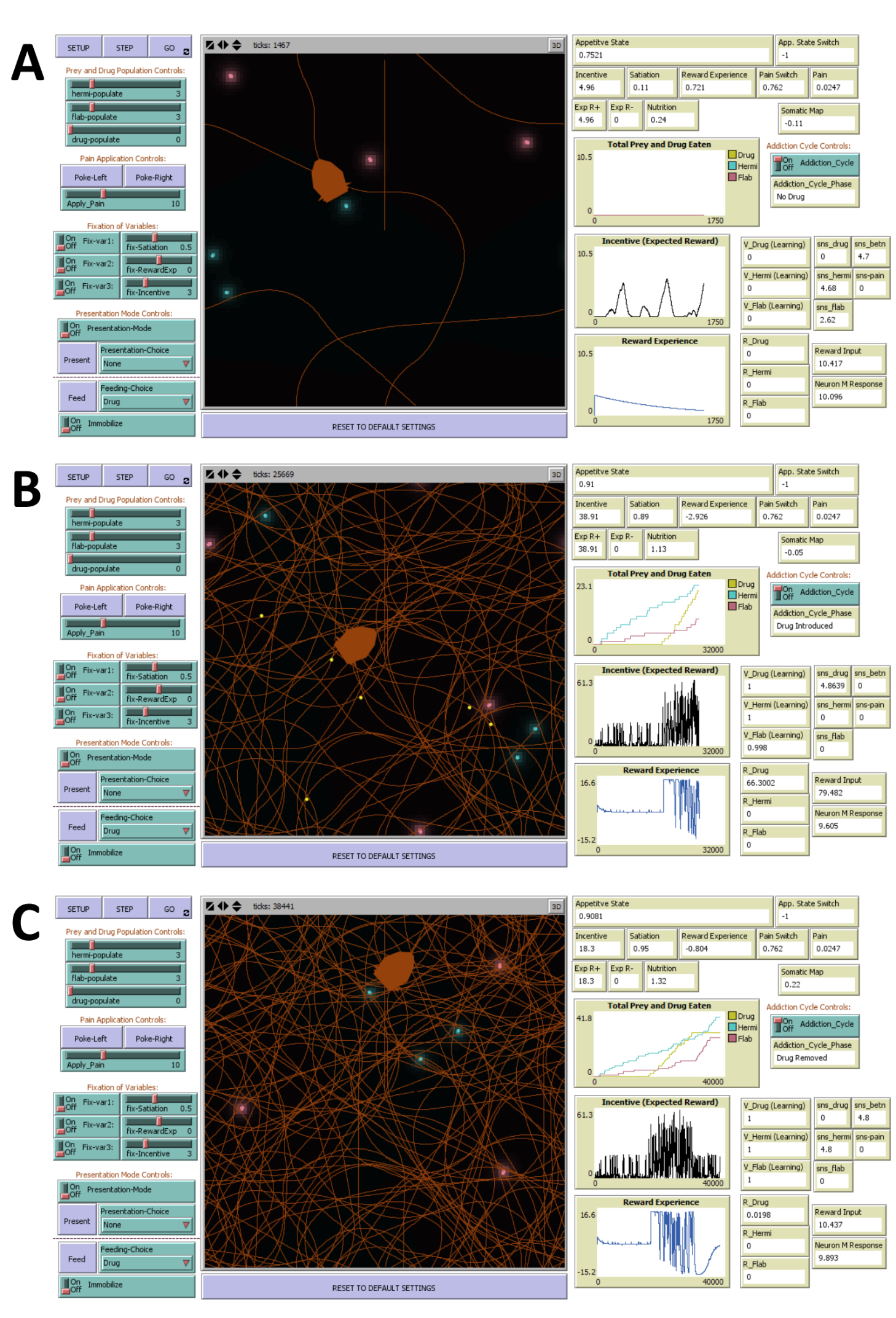
**

**Figure S1**. Screenshots of the ASIMOV environment and interface (NetLogo version 6.0.4; http://ccl.northwestern.edu/netlogo/). There are three user modes. In the default foraging mode, the forager (orange) encounters Hermi (blue orbs), Flab (pink orbs), and Drug (yellow orbs) and traces its path (orange contours). The user can select numbers of prey and Drug items in the environment and fix values of Satiation, Reward Experience, and Incentive. In Presentation Mode, the forager is immobilized and its prey and Drug intake are controlled to monitor approach and avoidance responses. Pain stimuli of adjustable magnitude can be applied to the left or right side of the ASIMOV agent’s head to test the turn response. On the right of the interface, Addiction Cycle Mode, when enabled, allows the forager to forage freely and experience different phases of addiction in a dynamic environment. Drug availability changes over time, causing the forager to experience addiction, desensitization, withdrawal, and cravings (see text). A) ASIMOV’s agent forages in the No Drug phase of the Addiction Cycle. Note that it initially performed several avoidance turns, and at the moment of the screenshot it is making an approach turn towards a prey. B) ASIMOV’s agent forages in the Drug Introduced phase of the Addiction Cycle. Drug consumption significantly increases the forager’s reward experience. As ASIMOV’s agent continues Drug consumption, there is desensitization to the reward received from the Drug, and thus more fluctuation in reward experience, as seen in C. C) In the Drug Removed phase of the Addiction Cycle, without Drug access ASIMOV’s forager undergoes withdrawal, represented in negative reward experience.

**Supplementary References**

1 Brown, J. W. *et al.* Implementing Goal-Directed Foraging Decisions of a Simpler Nervous System in Simulation. *eNeuro* **5**, ENEURO. 0400-0417.2018 (2018).

2 Wilensky, U. NetLogo: Center for connected learning and computer-based modeling. *Northwestern University, Evanston, IL* **4952** (1999).

3 Noboa, V. & Gillette, R. Selective prey avoidance learning in the predatory sea slug *Pleurobranchaea californica*. *J. Exp. Biol.* **216**, 3231-3236 (2013).

4 Gillette, R., Huang, R.-C., Hatcher, N. & Moroz, L. L. Cost-benefit analysis potential in feeding behavior of a predatory snail by integration of hunger, taste, and pain. *Proc. Nat. Acad. Sci. USA* **97**, 3585-3590 (2000).

5 Rescorla, R. A. & Wagner, A. R. A theory of Pavlovian conditioning: Variations in the effectiveness of reinforcement and nonreinforcement. *Classical conditioning II: Current research and theory* **2**, 64-99 (1972).

6 Berridge, K. C. & Robinson, T. E. Liking, wanting, and the incentive-sensitization theory of addiction. *Am. Psychol.* **71**, 670 (2016).

7 McSweeney, F. K. & Murphy, E. S. Sensitization and habituation regulate reinforcer effectiveness. *Neurobiol. Learn. Mem.* **92**, 189-198 (2009).

8 Cabanac, M. Sensory pleasure. *The quarterly review of biology* **54**, 1-29 (1979).
